# Solitary, Late Metastatic Recurrence of Renal Cell Carcinoma to the Pancreas: A Case Report

**DOI:** 10.7759/cureus.8521

**Published:** 2020-06-09

**Authors:** Khalil Choucair, Nathaniel A Parker, Ammar Al-Obaidi, Joel Alderson, Phu Truong

**Affiliations:** 1 Internal Medicine, University of Kansas School of Medicine, Wichita, USA; 2 Pathology, Ascension Via Christi St. Francis Hospital, Wichita, USA; 3 Hematology/Oncology, Cancer Center of Kansas, Wichita, USA

**Keywords:** carcinoma, renal cell, clear renal cell carcinoma, metastasis, pancreatic metastasis, late recurrence

## Abstract

Renal cell carcinoma (RCC) accounts for 3% of all adult malignancies and is known for metastatic initial presentation, unpredictable metastatic pathway, and late recurrence post-curative resection. We report a case of solitary late metastatic renal cell carcinoma to the pancreas more than 10 years after radical nephrectomy. A high index of suspicion must be maintained to detect RCC late recurrence and metastasis to rare and atypical locations. A lifelong follow-up is recommended.

## Introduction

Renal cell carcinoma (RCC) is the most common type of kidney cancer in adults, and accounts for around 3% of adult malignancies [1]. While radical nephrectomy is the standard of care for localized primary RCC, up to one-third of RCC initially present with metastatic disease [2]. However, even those presenting with a localized disease, eventually metastasize despite the curative procedure [3]. In fact, RCC is known to have an unpredictable metastatic pathway, and late recurrence is characteristic of this tumor. Arbitrarily, late recurrence describes a recurrence that develops more than 10 years after nephrectomy [4, 5], and incidence of this phenomenon has been reported to range between 4.3% and 11% of cases [5, 6]. RCC most commonly metastasizes to the lungs, lymph nodes, bone and liver [2]. Metastasis to the pancreas accounts for less than 5% of all pancreatic malignancies [7]. RCC specifically, only accounts for 0.25% to 3% of all resected pancreatic masses [8-10]. Here, a rare case of a patient with RCC who developed late, solitary metastasis to the pancreas is presented.

## Case presentation

**Figure 1:**
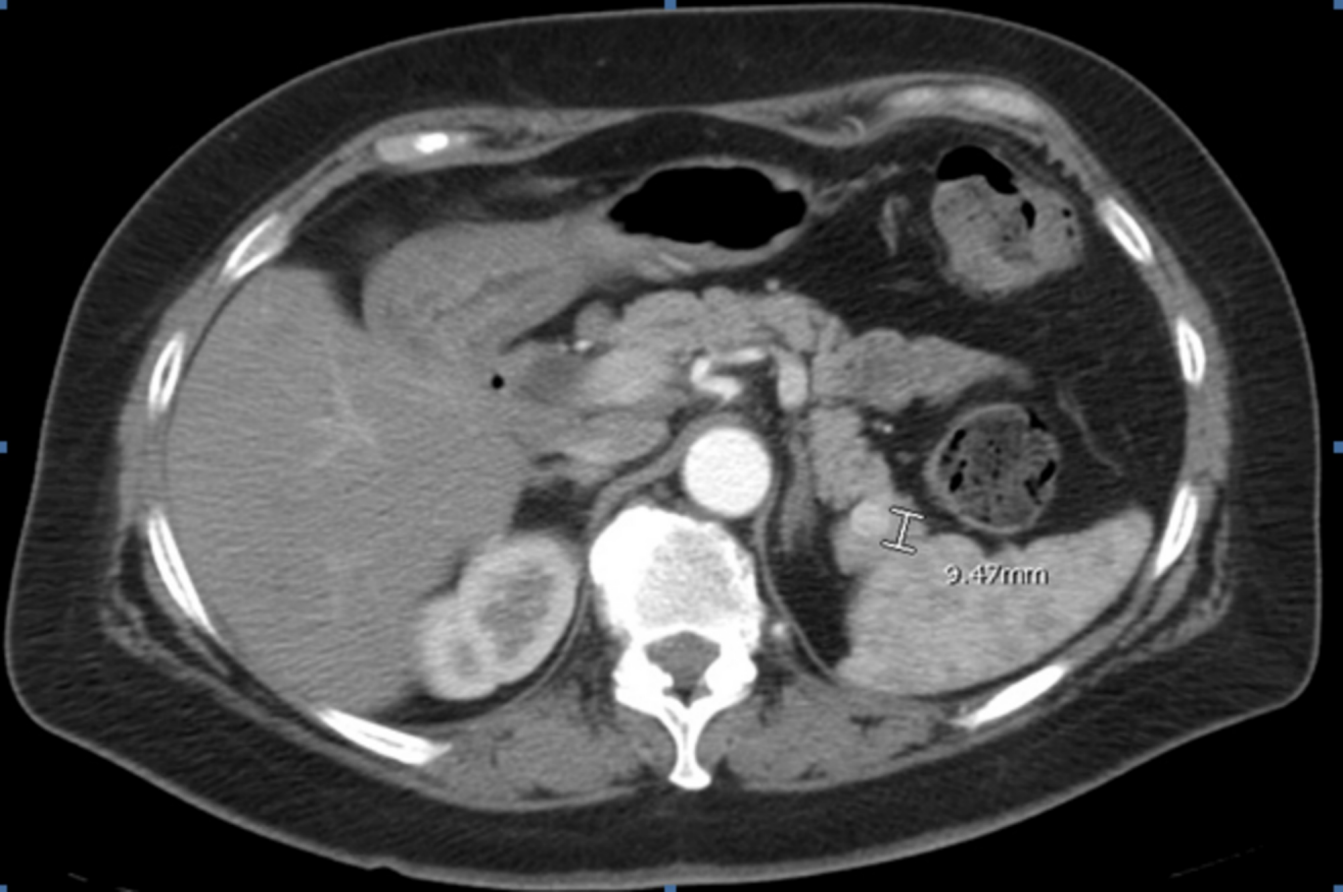
Year-nine post-nephrectomy imaging showed a single pancreatic tail lesion. CT of the abdomen and pelvis demonstrated a hypervascular focus demonstrated within the distal aspect of the pancreas measuring 0.9 cm. At that time, the enhancing focus within the tail of the pancreas appeared of the same density as the mesenteric arteries on that particular examination and was suspected to reflect a small splenic artery aneurysm.

**Figure 2:**
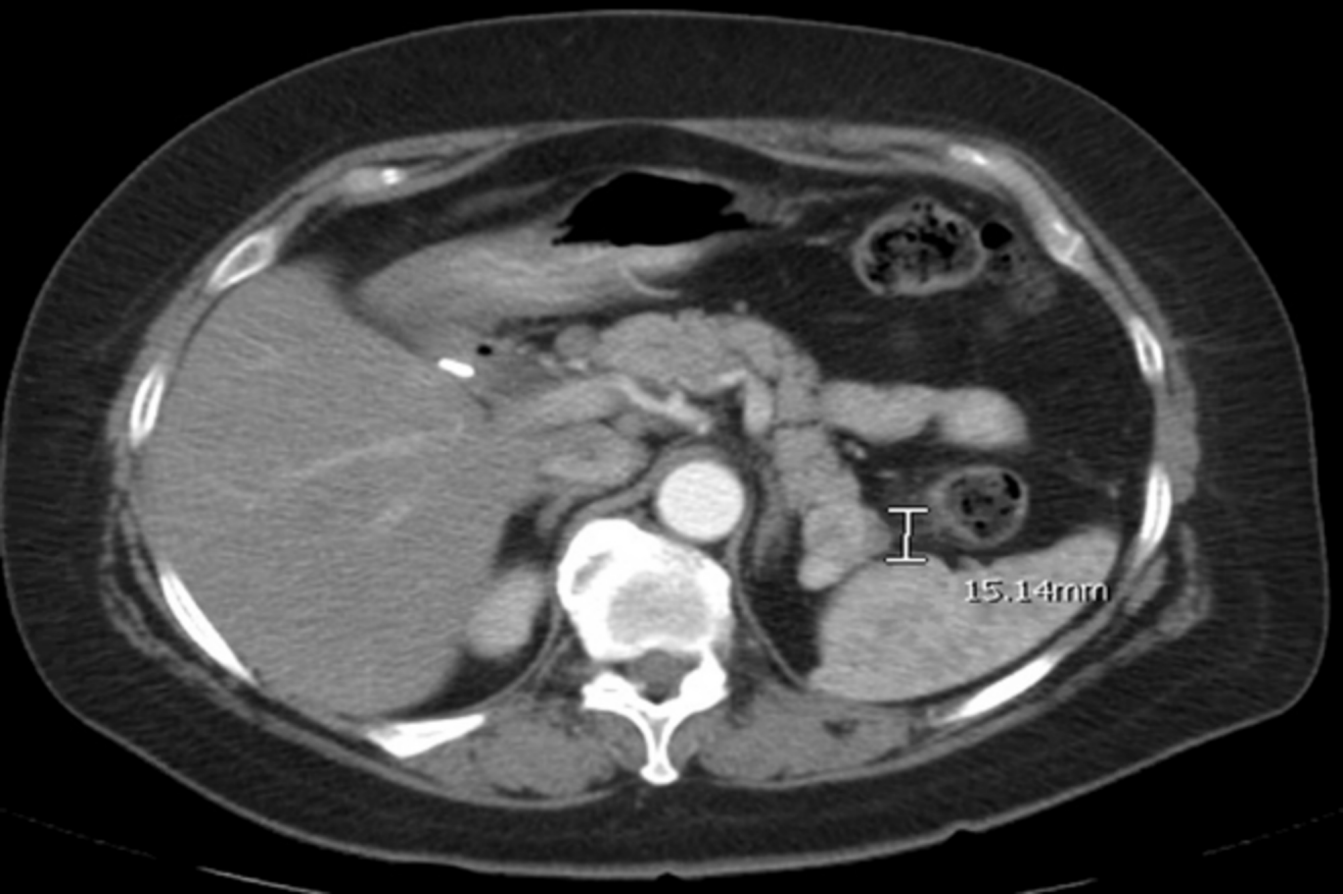
Year-eleven post-nephrectomy imaging showed interval increase of the pancreatic tail lesion. CT abdomen and pelvis demonstrated a mild increase in size of the mass lesion in the pancreatic tail. The lesion measured 0.9 cm previously, experienced an interval increase in size, and then measured about 1.5 cm.

**Figure 3:**
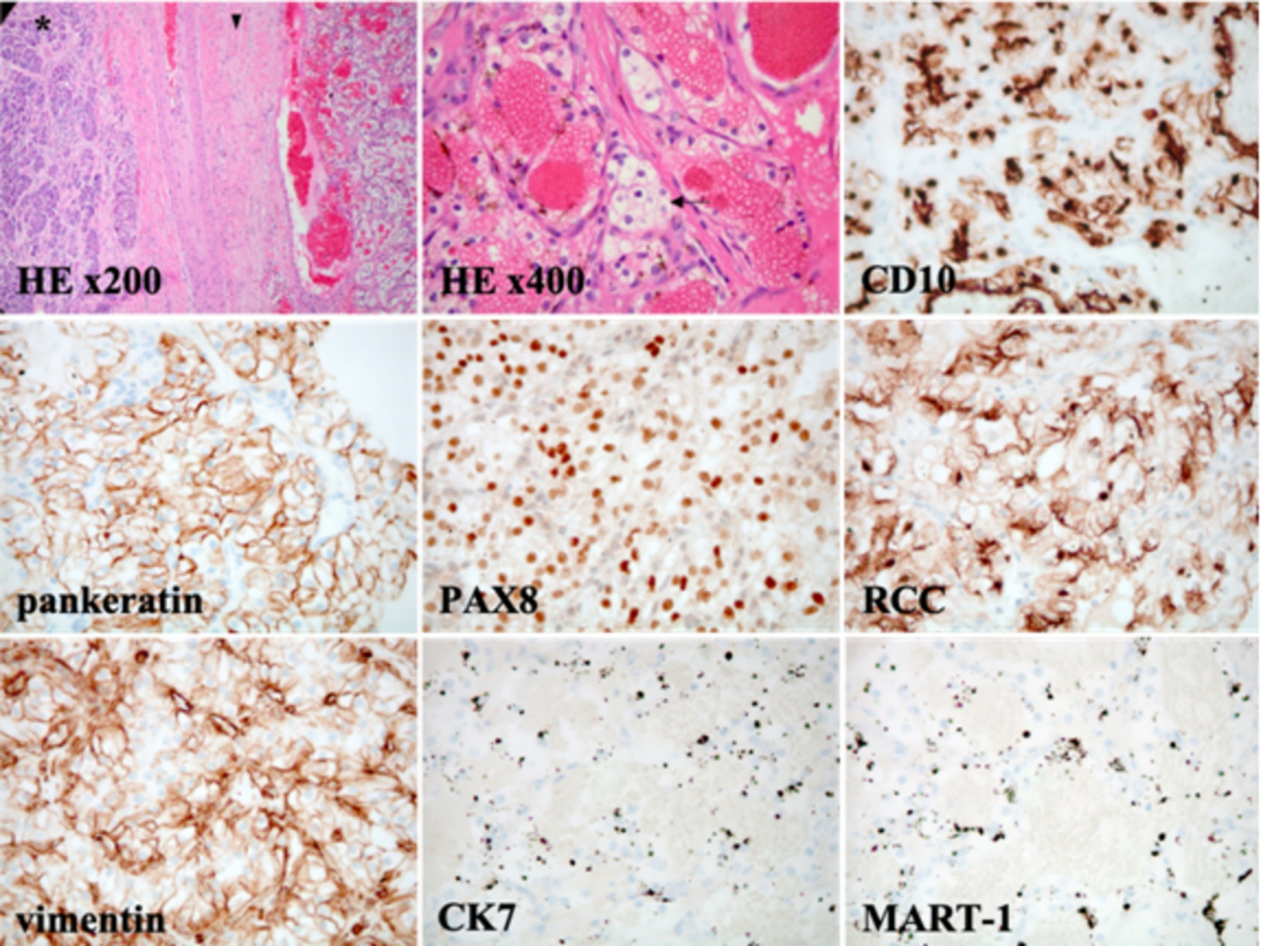
Pathology demonstrates the pancreas with metastatic low-grade cRCC. At medium power magnification (HE x200), malignant tissue is somewhat encapsulated (arrowhead) intervening with capillaries and adjacent to normal-appearing pancreatic tissue (asterisk). Histopathology reveals the malignant cells are rounded with small nucleoli and clear cytoplasm (arrow) arranged in lobules (HE x400). At high power magnification (x400), tumor cells stained with CD10, pankeratin, PAX8, RCC, and vimentin by IHC are positive, whereas CK7 and MART-1 (melan-A) are negative. Ultimately, histopathology and IHC demonstrates features compatible with a low-grade, poorly differentiated cRCC.

A 61-year-old female presented to her oncologist for routine surveillance monitoring. She had a past medical history notable of left clear renal cell carcinoma (cRCC; T2aN0M0) treated with radical left nephrectomy, a solitary 0.9 cm pancreatic tail mass noted on year-nine post-nephrectomy surveillance imaging, and benign bilateral primarily sub-centimetric lung nodules (Figure 1). Of note, these pulmonary nodules had remained the same size compared to prior imaging studies and determined to be positron emission tomography (PET)-negative. At year 11 post-nephrectomy, routine surveillance by contrasted abdominopelvic CT scans revealed interval growth of the pancreatic tail mass from 0.9 cm to 1.5 cm, compared to previously stable prior monitoring scans (Figure 2). She underwent endoscopic ultrasound, which revealed a hypoechoic pancreatic tail mass, measuring 1.8 x 1.4 cm. Fine needle aspiration of the mass was negative for malignant cells. Also, cytologic analysis was unable to identify *KRAS* mutations. Subsequent robotic partial pancreatectomy and splenectomy was performed and provided the final pathological diagnosis. Grossly the pancreatic mass was found to be 2.3 x 2.0 x 1.4 cm in size. Surgical margins were negative and the 14 resected regional lymph nodes were negative for malignancy. While the spleen was found to harbour no malignancy, tumor cells from the pancreatic mass resembled clear cell-type RCC. Tumor cells showed immunoreactivity with CD10, pankeratin, PAX8, RCC, and vimentin markers, but were negative for CK7 and MART-1 staining (Figure 3). Cytology was repeated and molecular characterization revealed wild type *KRAS* status. Together with imaging, surgical specimen histopathology, and this immunohistochemical staining profile isolated and metastatic cRCC was diagnosed in year-eleven post-nephrectomy.

The patient underwent complete pancreatectomy, which intraoperatively revealed no other concerning masses or lymphadenopathy. She was started on combination immunotherapy consisting of nivolumab plus ipilimumab, and followed to monitor for treatment response and resolution. Routine surveillance monitoring by PET/CT scans at standard-of-care intervals has shown complete resolution. This is consistent with a durable and long-lasting treatment response for this rare and late recurrence of cRCC despite presumed definitive surgical management by nephrectomy almost a decade prior. The patient remains alive, healthy, symptom-free, and in complete remission to this day, approximately one year since initiating combination immunotherapy.

## Discussion

We have reported a relatively rare case of RCC recurrence involving the pancreas, an unusual site of RCC metastasis. Although RCC usually metastasizes to the lungs (76%) and lymph nodes (66%) most frequently, followed by bone (42%) and liver (41%) [2, 11], it can also metastasize to unusual sites, such as the pancreas. In fact, RCC only accounts for 0.25% to 3% of all resected pancreatic masses [8-10], and similar to our case, other reports of solitary pancreatic metastasis have been reported [10, 12-14].

Besides the peculiarity of the site of recurrence, RCC is notorious for distant metastasis with no clinical suggestion of a primary lesion, like in our case, and late recurrence, more than 10 years after curative nephrectomy, is one of the unusual characteristics of RCC [5, 6, 11]. Some studies have even reported recurrences as late as 30 and 45 years following initial surgery [15, 16]. Several theories have been proposed for delayed and distant metastasis in RCC: decreased host immunity that allows uncontrolled growth of tumor cells, or change in hormonal levels (since late recurrence is 2-3 times more frequent in women), has been speculated [17, 18]; furthermore, studies have proposed that RCC may shed tumor-derived microvesicles that disperse through hematogenous routes, and seed new distant sites [2].

Several studies have examined specific risk factors for this delayed recurrence: in a cohort consisting of 459 RCC cases treated by radical nephrectomy, Tellini et al. demonstrated that positive surgical margins were an independent predictor of recurrence with a higher incidence of distant and local relapses [19]. Similarly, in a study involving 1454 patients treated with nephrectomy for localized RCC, risk factors that correlated with increased risk of delayed recurrence included: increased tumor size, clear cell or collecting duct histological features, and increasing tumor staging [6]. Whether the molecular make up of the metastatic lesion is identical to the primary tumor remains unknown, it would certainly be interesting to further investigate. In fact, while histology is frequently used to prognosticate RCC patients, the era of next generation sequencing offers the possibility to characterize molecular markers of delayed recurrence, as well as metastasis site preference: alterations in mTOR pathway, SETD2, PTEN and KDM5C have been reported to variably occur across different sites of RCC metastasis [2]. Similarly, clonal origin of these delayed recurrences can potentially be assessed at a molecular level to detect the driving alterations behind such recurrences, and potentially target them.

Unlike many solid tumors that metastasize, surgical resection of single or limited number of metastases is a possible therapy in carefully selected mRCC patients, even in the setting of late metastatic recurrence after initial nephrectomy [14]. Whether adjuvant therapy, following surgical resection, offers survival advantage over surgery alone remains inconclusive based on the available data [20].

## Conclusions

Despite advances in therapeutic options, RCC remains one of the most aggressive malignancies with risk of delayed recurrence to some rare and atypical locations. Thus, and along with the established surveillance protocols, some lesions may still be missed especially when presenting in atypical locations and a high index of suspicion must be maintained.
